# Eating for 1, Healthy and Active for 2; feasibility of delivering novel, compact training for midwives to build knowledge and confidence in giving nutrition, physical activity and weight management advice during pregnancy

**DOI:** 10.1186/1471-2393-14-218

**Published:** 2014-07-04

**Authors:** Andrea Basu, Lynne Kennedy, Karen Tocque, Sharn Jones

**Affiliations:** 1 Betsi Cadwaladr University Health Board, Wrexham Maelor Hospital, Croesnewydd Road, Wrexham LL13 7TD, UK; 2 Department of Clinical Sciences & Nutrition, University of Chester, Parkgate Road, Chester CH1 4BJ, UK; 3 KT Intelligence CIC/University of Chester, Regus House, Herons Way, Chester Business Park, Chester CH4 9QR, UK

**Keywords:** Nutrition, Weight management, Physical activity, Obesity, Training

## Abstract

**Background:**

Women in Wales are more likely to be obese in pregnancy than in any other United Kingdom (UK) country. Midwives are ideally placed to explore nutrition, physical activity and weight management concerns however qualitative studies indicate they lack confidence in raising the sensitive issue of weight. Acknowledging this and the reality of finite time and resources, this study aimed to deliver compact training on nutrition, physical activity and weight management during pregnancy to increase the knowledge and confidence of midwives in this subject.

**Methods:**

A compact training package for midwives was developed comprising of evidence based nutrition, physical activity and weight management guidance for pregnancy. Training was promoted via midwifery leads and delivered within the Health Board. Questionnaires based on statements from national public health guidance were used to assess changes in self-reported knowledge and confidence pre and post training. Descriptive statistics were applied and 95% confidence intervals were calculated.

**Results:**

43 midwives registered for training, 32 (74%) attended and completed the questionnaires. Although, pre training knowledge and confidence varied between participants, statistically significant improvements in self-reported knowledge and confidence were observed post training. 97% indicated knowledge of pregnancy specific food and nutrition messages as ‘better’ (95% CI 85 to 100), as opposed to 3% stating ‘stayed the same’ – 60% stated ‘much better’. 83% indicated confidence to explain the risks of raised BMI in pregnancy was either ‘much’ or ‘somewhat better’ (95% CI 66 to 93), as opposed to 17% stating ‘stayed the same’. 89% indicated confidence to discuss eating habits and physical activity was ‘much’ or ‘somewhat better’ (95% CI 73 to 97) as opposed to 11% stating ‘stayed the same’. Emergent themes highlighted that training was positively received and relevant to midwifery practice.

**Conclusions:**

This study provides early indications that a compact nutrition, physical activity and weight management training package improves midwives self-reported knowledge and confidence. Cascading training across the midwifery service in the Health Board and conducting further studies to elicit longer term impact on midwifery practice and patient outcomes are recommended.

## Background

Women in Wales are more likely to be obese (Body Mass Index, BMI ≥ 30) in pregnancy than in any other UK country; findings from a national project reports prevalence of maternal obesity classes II (BMI ≥35) and III (BMI ≥ 40) are highest within Wales, affecting around 1 in 15 or 6.5% of all pregnancies [1]. Routine data gathered in 2012 by Maternity Services in the Health Board Region suggests a progressive trend with approximately 11.6% women having a BMI ≥35 and an overall 27% of women classified as obese (BMI ≥30) at the start of their pregnancy [2]. This presents a key public health concern as obese pregnant women and their babies experience significantly greater risks compared with women of a healthy BMI, including miscarriage, gestational diabetes, pre-eclampsia, caesarean sections, anaesthetic complications, wound infections, stillbirth, congenital abnormalities, macrosomia and neonatal death [3]. Obese mothers are less likely to breastfeed their infants [4], and children of obese parents are more likely to become obese adults [5].

In 2010 the Centre for Child and Maternal Enquires and Royal College of Obstetricians [3], and the National Institute for Health and Clinical Excellence (NICE) [6] issued guidance to prompt improvements in the prevention and management of maternal obesity. These responded to concerns highlighted within national reports including ‘Why Mothers die’ ‘Saving Mothers Lives’, and ‘Perinatal Mortality [7-9]. National guidance includes recommendations outlining the importance of measuring weight, height and calculating BMI at the initial antenatal assessment, discussing the risks associated with obesity in pregnancy, and enabling access to appropriate and accurate advice on nutrition, physical and weight management. However as one national survey, undertaken by Netmums and the Royal College of Midwives [10] reports, there is a perceived lack of opportunity to discuss nutrition or weight management concerns within antenatal appointments; 61% (n = 3749) women indicated they were not given time to talk about their concerns in this area. Furness and colleagues [11] identified a lack of confidence amongst obese pregnant women regarding what foods they should eat, how much and what types of exercise are safe, and how much weight gain is acceptable. Furthermore evidence is evolving to suggest that dietary quality decreases amongst women in the higher BMI categories [12,13]. It is widely acknowledged that pregnancy is a key life course stage at which to intervene, when women are more receptive to health messages [14,15], and is thus an opportunity to effectively engage women in discussions about lifestyle and weight management.

Notwithstanding this opportunity, Furber and colleagues [16] explored the experiences of women who were obese during pregnancy and identified particular sensitivity to their size; thus advising caution around interventions or interactions with health professionals, to minimise distress. Obese pregnant women are a vulnerable group who are likely to have a history of weight concerns and possible negative experiences with healthcare provision [17]. Consequently lifestyle messages should be approached respectfully, with advice offered neutrally and free from judgement [18].

Indications from qualitative research suggests midwives are not wholly confident to provide personalised nutrition or weight management advice to obese women during pregnancy [19-21]. This may negatively impact on their ability to routinely discuss the topics with women who have a raised BMI, as advocated by national public health guidance [6]. These findings highlight a need to facilitate educational opportunities for midwives to instil professional confidence, knowledge and skills in engaging with pregnant women on these issues. The value of Motivational Interviewing (MI) theory has been recognised within the Strategic Vision for Maternity Services in Wales [22]. Evidence suggests that health-related behaviour change based on MI can be effective [23,24] and therefore may assist midwives in engaging pregnant women in effective ‘*change talk’*, with the potential to result in positive lifestyle change. MI has been described as a “*collaborative conversation style for strengthening a person’s own motivation and commitment to change*” [25]. Opportunities to increase the knowledge and confidence of Midwives in addressing women’s concerns related to nutrition, physical activity and weight management could therefore seek to embed the foundations of MI.

It is widely accepted that midwives are a key professional group who are well placed to assist women with concerns related to nutrition, physical activity and weight management, however the profession is challenged by contemporary workforce issues. Under estimates of the projected birth rate have led to increased workloads which coupled with an ageing midwifery workforce, and more midwives opting to work part time hours [26] has presented significant pressures in terms of achieving safe clinical caseloads and effective workforce planning. Such pressures clearly impact on the ability of NHS organisations to release midwives from clinical duties and enable them to attend necessary training updates. Practising midwives require access to training that meets their professional needs both efficiently and effectively. Training models that can deliver on both these levels are vital for securing and embedding continuing professional development opportunities for midwives; particularly in light of the UK’s current economic climate and the challenges faced by public services.

Given the mounting evidence of identifiable risks linked with maternal obesity, NHS services are required to implement measures to manage these risks and improve outcomes for obese pregnant women and their babies. The Welsh Risk Pool Services [27] indemnifies NHS Health Boards in Wales in relation to formal claims for clinical negligence and personal injury, and assesses clinical standards specific to maternity services. This requires evidence of an approved, written policy, procedure, pathway or guidelines specific to management of women with obesity in pregnancy, and indicates that as part of the initial risk assessment all mothers should be offered advice about diet, exercise and weight management, alongside discussion of the associated risks of a raised BMI in pregnancy. Currently the UK has no guidelines for gestational weight gain which creates some difficulty for those advising women, and for women themselves to understand what is considered a safe and not excessive weight gain [11,14,28]. NICE [6] advise women should not be weighed repeatedly during pregnancy unless clinical management can be influenced or if nutrition is of concern, however no guidance is given on how weight should be monitored under these circumstances. The United States (US) have established evidence based guidelines [29] though limited high level evidence linking these with improved pregnancy and foetal outcomes has caused some contention with respect to their application in the UK [30]. However, the US guidelines have been deemed safe [31] and arguably provide a helpful reference whilst the evidence base continues to be strengthened.

Maternal obesity places a substantial social and economic burden on individuals, the NHS and society [32]; therefore interventions that can effectively equip midwives to competently and sensitively tackle this pressing public health issue are required.

## Methods

### Study design, setting and participants

This study was funded by the Health Board as an evaluative feasibility study based on simple experimental design (pre-and post-test). The aim was to explore the feasibility of delivering a compact and novel training model to increase the knowledge and confidence of midwives in giving nutrition, physical activity and weight management advice during pregnancy. This took place across a single but large Health board with a population (n = 676,000) accounting for approximately 20.2% (n = 7210) of live births in Wales [33,34]. Community midwives employed within the Health Board were invited to attend the compact training which was promoted through midwifery team leaders via an electronic flyer, and posters displayed within staffing areas. Community midwives were targeted as they are largely responsible for managing antenatal consultations, in particular the initial ‘booking’ appointment where height, weight, and BMI measurements should be taken, risks related to a raised BMI in pregnancy discussed, and guidance on nutrition and physical activity provided [6]. A total of 40 training places were available, this represents 41% of the total community midwifery workforce within the Health Board (n = 97). Training places were allocated on a first come first served basis, and were limited to 10 participants per session due to venue capacity, and a preference to maintain a high level of interaction amongst participants and with the training facilitator. In total 43 midwives registered to attend and all were offered a place. Registering midwives were not required to indicate their pay band, or years of midwifery experience, however general correspondence during the training process indicated the cohort consisted of community and integrated midwives at NHS pay bands 6 and 7. Some were relatively new to the profession and others approaching retirement. A total of 4 compact training sessions were facilitated across the Health Board during January 2013.

### The training model

From the outset maternity services specified that training on nutrition, physical activity and weight management would need to be delivered in a compact format due to competing training commitments and constraints of releasing staff from work duties. A half day (3.5 hours) was negotiated between the principal researcher (training facilitator) and head of women’s outpatient services as a realistic timeframe within which to deliver the training whilst minimising disruption and cost to the midwifery service.

Delivery took place within cost neutral venues, predominantly NHS owned. The training was facilitated by a registered dietitian who worked closely with midwifery services to develop overall content and structure.

The compact training model & learning materials developed for this study were informed by a scoping study guided by the principles of undertaking an integrative review [35]. This included;

• Identification of evidence based guidelines [3,6].

• Examination of published quantitative and qualitative studies focused on maternal obesity; women’s perspectives on care received; health professional’s reported knowledge, skills and confidence to advise women on aspects of nutrition, physical activity and weight management in pregnancy.

• Consultation with a registered psychologist/accredited psychotherapist/MI trainer, and published texts on MI theory [25,36].

• Communication with senior midwives and observation of community midwives delivering antenatal consultations.

• Communication with an exercise physiologist to clarify guidance on physical activity during pregnancy.

The learning outcomes prepared for the training model are outlined below:.

Learning Outcomes:

Participants attending the compact training will be able to:

• understand the risks of obesity in pregnancy

• understand maternal nutritional requirements for a healthy and safe pregnancy

• understand current recommendations for physical activity and exercise during pregnancy

• understand how to support women to maintain a healthy and safe weight during pregnancy

Prior to attending the compact training session participants were emailed a pre course reading list signposting them to specific sections of evidence based reports and guidelines that would be referenced throughout the training. To facilitate achievement of the learning outcomes a mix of teaching and learning methods were incorporated including group discussions, short lectures, activities using food models and worksheets. Brief conversational scripts were developed and filmed as video clips to facilitate discussions on raising the sensitive issue of weight during pregnancy, utilising key principles of motivational interviewing.

### Data collection

Pre (Additional file 1) and post (Additional file 2) training questionnaires were developed to identify the self-reported knowledge and confidence of midwives who attended and whether this was improved. Questions were based on statements extracted from recommendation two of the NICE guidance [6] which focuses on aspects of care for pregnant women with a BMI ≥ 30. Participants were asked to rate their knowledge and confidence for each of the statements prior to undertaking the training using a 10-point scale [37] with a rating of 1 indicating poor knowledge/not at all confident, and 10 indicating highly knowledgeable/extremely confident.

A 4-point and 5-point Likert scale [37] was used to measure changes in knowledge and confidence respectively for participants on completion of training. Participants were asked to consider each of the statements (the same as those presented in the pre training questionnaire), and select the option best suited to them; potential responses included;

My knowledge: is much better/is somewhat better/is a little better/has stayed the same.

My confidence: is much better/is somewhat better/has stayed the same/is somewhat worse/is much worse.

Supplementary questions were included within the post training questionnaire to identify participant’s satisfaction with the training. These included a mix of response options including free text, yes/no, and a 4-point scale, excellent/good/adequate/poor.

The questionnaires were reviewed by senior midwifery managers and an experienced epidemiologist (KT). They were then piloted in an independent maternity unit before being finalised. On arrival training participants were welcomed and invited to complete the pre training questionnaire; similarly participants were thanked for attending the session at its conclusion and invited to complete the post training questionnaire prior to leaving. At both these time points the trainer explained the questionnaires were designed to enable evaluation of the training, identify any improvements should the training be replicated in the future, and to provide overall feedback to the midwifery service in the Health Board. The trainer informed and reassured participants that the questionnaire was anonymous and could be completed voluntarily. Consent was then implied by those who chose to complete the questionnaire after providing the above information. Both the pre and post training questionnaires were included in a training pack given to each participant on arrival. Each pack was coded e.g. 001, 002 etc. to enable pairing of pre and post training questionnaires for data analysis. Participants were directed to where the questionnaires were located in their packs, and if willing to complete they were asked to leave their completed forms either where they had been sitting or hand them to the trainer upon leaving.

### Data analysis

Responses were entered onto a spreadsheet, to analyse the data using basic descriptive statistics (median, range) and probability. With respect to the post course questionnaire, to increase the statistical power of the categories each scaled answer was grouped into two categories ‘Better’ (which included ‘much’, ‘somewhat’ and ‘a little’ better) and ‘Stayed same’. The Clopper-Pearson exact method was used to calculate 95% Confidence Intervals (CI’s) for proportions for each of the knowledge and confidence questions. Any CI’s that were not overlapping were considered to be statistically different. Levels of knowledge and confidence pre-training were analysed using the nonparametric Friedman’s analysis of variance test for related samples, followed by a post-hoc Wilcoxen tests with Bonferoni correction (using SPSS v. 21).

### Ethics

The study did not involve NHS patients and was classified as audit and evaluation, not research therefore full or proportionate ethics via the Integrated Research Application System (IRAS) was not required. To help determine whether the study was classified as research we referred to the Health Research Authority tool [38]. The R&D office within the Health Board was contacted to formally register the study. As a service evaluation full ethics application was not required however the ethical aspects of undertaking the study were considered and governance guidance adhered to; we were conscious that health professionals in receipt of the training may feel obliged to participate in evaluation; we emphasised they were not obliged to participate in the evaluation and this would not affect their right to attend. Throughout the training participants were reminded of requirements to protect anonymity and confidentiality of clients.

## Results

In total, 32 community midwives (74% of those who registered) attended the compact training sessions. This represents 33% of the total community midwifery workforce employed within the Health Board. Of the 32 community midwives, some (n = 5) had an ‘integrated’ role which combines both hospital and community based midwifery duties. All participants voluntarily chose to complete both the pre and post training questionnaires.

### Pre training

Participants were asked to provide details of any previous learning undertaken on the topic areas of nutrition, physical activity, and weight management in pregnancy. More than half of the midwives (59%, n = 19) indicated that previous knowledge of the topic area was limited. A small number cited personal reading (n = 6), attendance at a local public health meeting (n = 3), information during pre registration training (n = 4), or completion of a specific course e.g. diabetes course, bio medical course, or aqua natal training.

Self-reported pre training knowledge and confidence levels varied across the range of statements (questions). Some participants selected ratings as low as 1 indicating they had poor knowledge and/or were not at all confident in relation to some of the statements. Conversely, other participants considered themselves as being highly knowledgeable and/or highly confident and selected ratings as high as 10 for some statements. Since levels of improvement will be dependent on existing levels, it was considered important to determine which statements scored the highest. Overall, the median scores were significantly different between statements, confirming that knowledge and confidence varied statistically between topics. Post-hoc pairwise comparisons (analysis not shown) indicated that only knowledge statement (d) and confidence statement (a) were consistently different from all other statements (Tables 1 and 2). This indicates that pre training knowledge of ‘benefits of being physically active during pregnancy’ and confidence to ‘measure weight and height at the first contact with pregnant women’ were significantly higher than for all the other questions.

**Table 1:** Participants pre and post training self-reported knowledge ratings & statistical significance

	**Pre training**	**Post training**	
**Knowledge of:**	**Average, median knowledge rating (response range)**	**% Participants rating knowledge as better - including ‘much’, ‘somewhat’ or ‘a little’ (% stating ‘much better’)**	**Statistical significance of a change in knowledge, yes/no 95% CIs expressed as (much, somewhat, a little better LCL to UCL; stayed the same LCL to UCL)**
	**1 = poor knowledge 10 = highly knowledgeable**		
(a) Range of risks related to obesity	8 (response range 1–10)	89% (40%)	Yes (73 to 97; 3 to 27)
(b) Pregnancy specific food and nutrition messages (based on the eatwell plate)	6 (response range 1–10)	97% (59%)	Yes (85 to 100; 0 to 15)
(c) Vitamins recommended during pregnancy, particularly for women with a raised BMI (including why, when, and amounts)	7.5 (response range 2–10)	80% (34%)	Yes (63 to 92; 8 to 37)
(d) Benefits of being physically active during pregnancy	8 (response range 4–10)	77% (31%)	Yes (60 to 90; 10 to 40)
(e) Recommended weight gain for women during pregnancy	7 (response range 2–10)	91% (69%)	Yes (77 to 98; 10 to 40)
(f) Ways to initiate conversations with women about ‘change’ related to their dietary and physical activity behaviours	7 (response range 1–10)	91% (60%)	Yes (77 to 98; 2 to 23)
**Statistical significance of difference in knowledge between statements** Friedman’s two-way ANOVA	P < 0.005		

**Table 2:** Participants pre and post training self-reported confidence ratings & statistical significance

	**Pre training**	**Post training**	
**Confidence:**	**Average, median confidence rating (response range)**	**% Participants rating confidence as better - including ‘much’ or ‘somewhat’ (no respondents stated confidence was worse)**	**Statistical significance of a change in confidence, yes/no**
	**1 = poor knowledge**		
	**10 = highly knowledgeable**		**95% CIs expressed as (Much better & somewhat better LCL to UCL; stayed the same LCL to UCL)**
(a) ‘measure weight and height at the first contact with pregnant women’	10 (response range 5–10)	40%	No (24 to 58; 42 to 76)
(b) ‘…being sensitive to any concerns she [the women] might have about her weight’	8 (response range 3–10)	77%	Yes (60 to 90; 10 to 40)
(c) ‘Explain to women with a booking appointment BMI of 30 or more how this poses a risk, both to their health and the health of the unborn child’	8 (response range 1–10)	83%	Yes (66 to 93; 7 to 34)
(d) ‘Explain that they should not try to reduce this risk by dieting while pregnant and that the risk will be managed by the health professionals caring for them during their pregnancy’	7 (response range 2–10)	89%	Yes (73 to 97; 3 to 27)
(e) ‘At the earliest opportunity…discuss her eating habits and how physically active she is. Find out if she has any concerns about diet and the amount of physical activity she does and try to address them’	8 (response range 1–10)	89%	Yes (73 to 97; 3 to 27)
(f) ‘Advise that a healthy diet and being physically active will benefit both the woman and her unborn child during pregnancy…Advise her to seek information and advice on diet from reputable sources’	7 (response range 3–10)	80%	Yes (63 to 92; 8 to 37)
(g) ‘Dispel any myths about what and how much to eat during pregnancy’	8 (response range 2–10)	83%	Yes (66 to 93; 7 to 4)
(h) ‘Offer practical and tailored information. This includes advice on how to use Healthy Start vouchers to increase the fruit and vegetable intake of those eligible…’	7 (response range 2–10)	80%	Yes (63–92; 8–37)
**Statistical Significance of difference in confidence between statements** Friedman’s two-way ANOVA	P < 0.005		

### Post training knowledge

**Figure 1:**
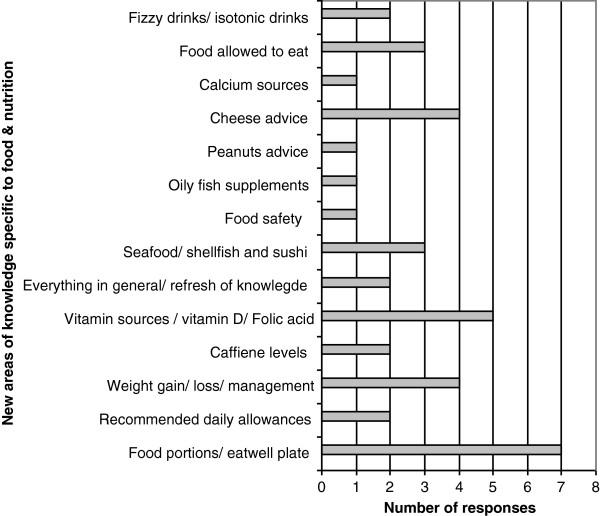
Number of participants citing new areas of knowledge specific to food and nutrition.

Statistically significant improvements in self-reported knowledge were observed for all questions a-f (Table 1). Improvements related to pregnancy specific food and nutrition messages were particularly high with 97% of participants rating their knowledge as ‘better’ following completion of the training; 40% stating knowledge was ‘much better’. A supplementary question asked participants to indicate if they had learnt anything new about food and nutrition that they hadn’t known before the training; 94% (n = 30) answered ‘yes’ which correlates with the observed improvement in knowledge specific to pregnancy food and nutrition messages. Where participants answered ‘yes’ they were prompted to give one or two examples. Specific detail about new information learnt is illustrated within Figure 1. A broad range of food and nutrition areas were identified, potentially indicative of the variance between participants in terms of their pre training knowledge. More popular responses included information specific to the eatwell plate (national food model), food portion sizes, and vitamin sources.

A similar degree of improvement was indicated for self reported knowledge related to recommended weight gain for women during pregnancy; 91% cited knowledge as being better, with 69% saying it was ‘much better’ post training. This finding was not entirely surprising given the absence of UK based clinical guidelines for gestational weight gain [6,11,14,28].

The lowest percentages with improvements in knowledge tended to be where prior knowledge (pre training rating) was highest.

### Post training confidence

None of the midwives in this cohort indicated a decrease in confidence following training. Statistically significant improvements in confidence were observed for questions b-h (Table 2), indicating participants felt more confident in raising the sensitive issue of weight, discussing the risks associated with a raised BMI, and reassuring women about safe approaches to eating well and being physically active during pregnancy.

Confidence intervals overlapped for question 3a which explored changes in confidence levels related to measuring weight and height at the first antenatal contact. 60% of the midwives trained reported their knowledge had ‘stayed the same’. However, pre training confidence in this was significantly higher (median = 10) than for all other statements, so it is not surprising to observe less improvement here. It’s possible that the 40% of midwives in this cohort who reported an increase in confidence for this question (not statistically significant) may have been less experienced community midwives.

### Participant satisfaction with training

Supplementary questions included within the post training questionnaire provided a measure of participant’s satisfaction with the training. Participants were asked was there anything not included in the training which should have been; 28% stated no (n = 9), and the remaining participants gave no response suggesting there were no obvious omissions, or areas which participants felt strongly should have been covered. When asked overall how they would rate the training, selecting a response from a 4-point scale (excellent/good/adequate/poor), 81% (n = 26) rated as excellent and the remaining 19% (n = 6) rated it as good. Furthermore when asked if they would recommend the training to others indicating with a simple yes/no response; all stated yes. A final question enabled participants to comment freehand on any other aspects of the training, responses here were grouped using the principles of thematic analysis [39].

Good Quality

• *“A very well presented study afternoon”*

• “*Excellent presentation and delivery*”

Valued Resources

• *“I will use the visual aids i.e. food plate”*

• *“Great folder with useful tips for women”*

Relevant to Midwives

• *“This covered many of the aspects of patient care we are responsible for”*

• *“Would be beneficial for all midwives and also valuable for student midwives”*

Much to fit in

• *“Really enjoyed but a lot to discuss in half a day”*

• “*Having a whole day would allow us to explore areas in more detail”*

## Discussion

This study explored the feasibility of delivering compact training on nutrition, physical activity and weight management to midwives. It was evident that midwives expressed knowledge and confidence prior to undertaking the training were significantly variable, despite this, early indications show statistically significant improvements in self-reported knowledge and confidence can be achieved when delivering a short, intense form of training.

### Knowledge

The most notable area of knowledge improvement related to pregnancy specific food and nutrition messages. This statement yielded the lowest pre training knowledge rating (median = 6) and the largest proportion of self-assessed improvement in knowledge post training (97%). Overall, the average pre-training self-reported knowledge scores were high (no statements had a median lower than 6) however, post training improvements in knowledge were statistically significant across all statements. This suggests the training was successful in improving the knowledge levels of participating midwives. Reported levels of knowledge pre training were on average higher than anticipated. This might be attributed to apprehension from participants in admitting to lower levels of knowledge on a subject that they give some advice about as a fundamental part of their role, or perhaps they felt self conscious in completing the questionnaire amongst other colleagues. Furthermore, they may have initially been unaware of potential gaps in their knowledge, as reflected within the four stages of learning [40].

Ultimately the desired impact of the training, from an observed enhancement in midwives knowledge is improved advice and guidance for pregnant women to help facilitate positive behaviour change. Qualitative evidence has indicated that pregnant women feel they have limited opportunities to discuss nutrition or weight with their midwife and may be confused about foods they should eat, exercise that is safe for them and their baby, and weight they should gain [10,11]. How effectively the improved knowledge is conveyed from midwives to women falls outside the scope of this study; further studies examining the wider and longer term impact of this training model and the potential for leading to positive lifestyle change amongst pregnant women is warranted.

Knowledge of recommended weight gain in pregnancy was also notably improved post training, however the guidance conveyed to midwives within this training model was based on the US Institute of Medicine’s (IOM) guidelines [29]. An absence of UK guidance on gestational weight gain has previously been noted to pose a significant barrier for midwives when advising women on what would be an appropriate weight gain [11,14,28]. The decision to incorporate US guidelines into the training model was debateable given a call for more robust, high level evidence linking these guidelines with significantly improved maternal and foetal outcomes [30]. However the IOM guidelines have been deemed safe and there is a valid argument for having such guidance as a reference, particularly in an attempt to minimise excessive weight gain, and minimise the risk of post-partum weight retention [31].

### Confidence

National guidelines [3,6] specify all pregnant women with a booking BMI ≥30 should be provided with accurate information about the risks associated with a raised BMI and how these may be minimised. Evidence from qualitative studies consistently identifies a lack of confidence amongst midwives in raising the sensitive issue of weight with pregnant women [11,14,15,18,20]. Midwives in this study rated their pre training confidence as relatively high (median = 8) with respect to explaining maternal obesity risks; a finding that was not anticipated given the consistency of the study findings noted above. Similar to the reported levels of pre training knowledge, this finding may be associated with trepidation amongst participants in admitting to a lack of confidence within a questionnaire they completed in the presence of colleagues or indeed on a topic where there is some expectation that this is a fundamental aspect of care they should provide. Despite this, a statistically significant improvement in confidence was observed in the post training responses. Confidence, when broaching the topic of weight and associated risks can be linked with uncertainty regarding the most appropriate approach to take once the risks are discussed [21], for instance what advice to offer, and where to signpost women to for further support. A considered strength of the compact training model was that it included learning activities specific to raising the issue and talking about weight, alongside recommendations and strategies that midwives could draw upon to advise women of the benefits of eating well and being physically active during pregnancy. Principles of motivational interviewing theory were integrated within the training model to facilitate helpful and non-judgemental discussions on nutrition and physical activity behaviours in an attempt to nurture the potential for stimulating positive lifestyle change. Excluding statement (a), post training improvements in confidence were evident across the range of given statements, suggesting the confidence gained was holistic and not restricted to just one aspect of a consultation centred around nutrition, physical activity and weight management. The non significance observed for statement (a), confidence to measure height and weight at the first contact with pregnant women, was not particularly surprising given present guidelines and reporting requirements which specify these measurements should be taken and recorded as standard [27,41].

### Replication of the training and application to practice

The quality and standard of the compact training was highly regarded by midwives within this study, with 100% rating excellent or good. The half day allocated for the training was regarded as limited given the breadth of content and complexity of some material. Although more time would be desirable this is not practical due to resource constraints on midwifery services and current requirements for mandatory training. The training facilitator was acutely aware of the challenge the agreed model presented in term of compressing the relevant information and learning materials into a compact training package. However, given the expressed improvement in knowledge and confidence of the topics covered, and overall satisfaction with the training received, this model could be regarded as a feasible means of up skilling midwives. Opportunities to refine the content of the training model including use of blended or online learning, could be explored and the potential influence and impact of these findings on routine practice warrants further exploration. Whilst this study was able to illustrate a positive shift in the knowledge and confidence of midwives, the ability to apply new knowledge, consolidate and build on their confidence is yet to be demonstrated. A recent study highlighted barriers to discussing the growing number of public health issues within antenatal consultations, expressing concern that this may simply promote a ‘tick box approach to care’ [42]. Assessing if and how midwives incorporate new knowledge into antenatal consultations is an important next step, as is identification of what may help or hinder midwives when attempting to integrate into practice. A follow up questionnaire could assist with this. Part of the compact training model encouraged midwives to engage in reflective activity post training. Templates were provided to assist reflective documentation following antenatal consultations where weight is discussed. Though this activity was not formally monitored post training, reflection on practice is recognised as good professional practice [43].

### Integration into pre and post registration midwifery education

To enable both future and practising midwives to acquire the necessary knowledge, and confidence in raising the issue of weight and effectively discussing nutrition, physical activity and healthy weight management, pre- registration midwifery education programmes and post registration continuing professional development should include all elements. A recent survey of Higher Education Institutions providing pre-registration midwifery education across the UK [42] highlighted specific gaps in obesity and nutrition education. Opportunities to engage with education providers to review curriculum needs, and explore integration of these key public health topics is strongly advocated. The compact training model developed here has potential to be integrated within pre registration midwifery curriculum and inclusion within an annual mandatory training programme for practicing midwives.

Nutrition is a fast moving science and aspects of nutritional guidance for pregnancy frequently changes, therefore health professionals with a responsibility for advising women need currency to ensure women receive accurate and consistent advice. Registered dietitians are well placed to support midwives in providing nutrition education, since they are uniquely qualified to use current public health and scientific research on food, health and disease, and interpret it into practical guidance to enable others to make informed lifestyle and food choices [44].

As adult obesity trends within Wales continue to rise [45], the number of women entering pregnancy with a raised BMI is also likely to increase. Opportunities to optimise weight prior to pregnancy is preferred, however approximately one third of pregnancies are unplanned [46], therefore NHS staff and services must be equipped to support pregnant women with a raised BMI to minimise risks to themselves and their babies. Health Boards in Wales are responsible for implementing measures to minimise risks for women and their babies [27], yet there is limited scope to reach midwives with training due to workforce pressures [26] and existing commitments for mandatory training. The compact training model described here offers a feasible solution; the potential to improve midwives knowledge and confidence on weight management during pregnancy in a way that is consistent with national guidance [3,6], yet with minimal disruption to, and loss of midwifery working hours. Furthermore, use of local NHS venues and professional expertise intrinsic to the NHS all contributes towards delivery of a cost efficient training model.

### Strengths and limitations

The content and delivery of the compact training model was informed by extensive exploration of historical and contemporary guidelines and evidence based practice, alongside detailed consultation with the midwifery profession, and experts working within the fields of MI and exercise physiology. This comprehensive approach ensured the training provided was of high quality and applicable to the midwifery profession.

Midwives expressed a high level of satisfaction with the training as evidenced by the post training responses. Interpretation of the self-reported post training questionnaire responses should acknowledge the possibility of a post course ‘feel good’ factor amongst those who participated. Also, as the questionnaires were administered by the trainer, social desirability may have influenced the results. However, the study does offer an early indication of the potential impact the training model has had on a small cohort of midwives. Due to the small sample size of midwives who attended the pilot training programme the response categories within the post course questionnaire had to be combined to enable statistical analysis to be performed. As further training courses are delivered a greater number of post training responses will be available to enable more robust analysis to be undertaken. Research which includes measurement of changes in professional’s knowledge of the subject would also be beneficial.

## Conclusion

Current prevalence of maternal obesity is concerning, as are the attributable maternal and foetal/neonatal risks. Pregnancy, a key stage in the life course, itself has considerations other than weight. Consequently, the NHS and its workforce have a duty of care to *sensitively* raise the issue of weight, namely for those women with a BMI ≥30; discuss the risks and reassure, support women to manage and minimise risks. Dietetic capacity within the NHS is limited and midwives are identified as an appropriate alternative source of advice. This study explored the feasibility of delivering novel, compact training to assist practicing midwives to tackle nutrition, physical activity and weight management concerns amongst pregnant women. Early indications are that community midwives who participated in the training experienced a statistically significant increase in knowledge and confidence on the subject matter; furthermore as a compact model it was found to be feasible for inclusion within existing resource constraints. Further research is required to examine the impact on midwifery practice, interactions with patients, and how this may affect clinical outcomes for women and their babies.

## Abbreviations

BMI: Body Mass Index; CMACE: Centre for Maternal and Child Enquiries; IOM: Institute of Medicine; MI: Motivational Interviewing; NICE: National Institute for Health and Care Excellence; WRPS: Welsh Risk Pool Services.

## Competing interests

The authors declare that they have no competing interests.

## Authors’ contributions

AB developed and delivered the compact training model. Prepared the draft manuscript, contributed to and coordinated manuscript revisions. LK provided supervision and guidance with development of the training, training questionnaires, data analysis and interpretation, and manuscript development, content and revisions. KT provided guidance on development of the training questionnaires, conducted work on data analysis and interpretation, and contributed to the original and revised manuscript. SJ facilitated liaison with midwifery services, provided maternity services data and engaged midwives to attend and participate in training, and commented on the original and revised manuscript. All authors read and approved the final manuscript.

## Authors’ information

AB (MSc BSc Hons RD) is a Registered Dietitian and Public Health Nutritionist who has worked in the field of public health nutrition within the NHS for over 13 years, she has a specific interest in maternal nutrition and provides nutrition education and training to a range of professional and community based workers.

LK (BSc (Hons), MPhil, PhD, AfN (PHN), Hon MFPH) is a registered Public Health Nutritionist, is Professor Public Health and Nutrition at the University of Chester. She has 25 years’ experience in academia and policy development in the field of nutrition, health promotion, nutrition epidemiology and public health nutrition.

KT (BSc PhD) is a trained epidemiologist and has over 25 years’ experience of statistics, data systems and research, with the last 18 years working in applied population and public health intelligence, with a specialist interest in health inequalities and population segmentation.

SJ (RN RM) is a practicing midwife with 23 years experience. She is currently the Head of Women’s Outpatient Services and has a passionate interest in public health issues which affect women.

## Pre-publication history

The pre-publication history for this paper can be accessed here:

http://www.biomedcentral.com/1471-2393/14/218/prepub

## Supplementary Material

Additional file 1 **Questionnaire (Pre training).** Study tool designed to determine self reported knowledge and confidence of midwives on the topic of nutrition, physical activity and weight management prior to attending the compact training. Click here for file

Additional file 2 **Questionnaire (Post training).** Study tool designed to determine changes in self reported knowledge and confidence of midwives on the topic of nutrition, physical activity and weight management after attending the compact training, and to elicit satisfaction with the training received. Click here for file
