# Supplementary material for: Eating for 1, Healthy and Active for 2; feasibility of delivering novel, compact training for midwives to build knowledge and confidence in giving nutrition, physical activity and weight management advice during pregnancy
Source: BMC Pregnancy Childbirth. 2014 Jul 4;14:218. doi: 10.1186/1471-2393-14-218 (PMC4227285; doi:10.1186/1471-2393-14-218)
Supplement: Additional file 1 — Questionnaire (Pre training). Study tool designed to determine self reported knowledge and confidence of midwives on the topic of nutrition, physical activity and weight management prior to attending the compact training. [file 1471-2393-14-218-S1.pdf]

Questionnaire (Pre Training)

1. **Please tell me about any previous learning you have undertaken on the topic areas of '*Nutrition, physical activity and weight management in pregnancy'*** (including pre and post registration training, CPD etc.)

2. **Take a moment to reflect upon your current knowledge of the following:**
For each question please circle the number that best describes your knowledge
1 = poor knowledge 10 = highly knowledgeable

(a) The range of risks related to obesity in pregnancy									
**1**	**2**	**3**	**4**	**5**	**6**	**7**	**8**	**9**	**10**
(b) Pregnancy specific food and nutrition messages (based on the eatwell plate)									
**1**	**2**	**3**	**4**	**5**	**6**	**7**	**8**	**9**	**10**
(c) Vitamins recommended during pregnancy, particularly for women with a raised BMI (including why, when, and amounts)									
**1**	**2**	**3**	**4**	**5**	**6**	**7**	**8**	**9**	**10**
(d) The benefits of being physically active during pregnancy									
**1**	**2**	**3**	**4**	**5**	**6**	**7**	**8**	**9**	**10**
(e) Recommended weight gain for women during pregnancy									
**1**	**2**	**3**	**4**	**5**	**6**	**7**	**8**	**9**	**10**
(f) Ways to initiate conversations with women about 'change' related to their dietary and physical activity behaviours									
**1**	**2**	**3**	**4**	**5**	**6**	**7**	**8**	**9**	**10**

3. **Now take a moment to reflect on how <u>confident</u> you <u>currently</u> feel to deliver on each of the statements in the boxes below** (extracted from NICE Guidance 27, 2010).
For each question please circle the number that best describes your confidence
1= not at all confident 10 = extremely confident

(a) 'Measure weight and height at the first contact with pregnant women'<br>**1 2 3 4 5 6 7 8 9 10**
(b) '… being sensitive to any concerns she [the woman] may have about her weight'<br>**1 2 3 4 5 6 7 8 9 10**
(c) 'Explain to women with a booking appointment BMI of 30 or more how this poses a risk, both to their health and the health of the unborn child'<br>**1 2 3 4 5 6 7 8 9 10**
(d) 'Explain that they should not try to reduce this risk by dieting while pregnant and that the risk will be managed by the health professionals caring for them during their pregnancy'<br>**1 2 3 4 5 6 7 8 9 10**
(e) 'At the earliest opportunity...discuss her eating habits and how physically active she is. Find out if she has any concerns about diet and the amount of physical activity she does and try to address them'<br>**1 2 3 4 5 6 7 8 9 10**
(f) 'Advise that a healthy diet and being physically active will benefit both the woman and her unborn child during pregnancy....Advise her to seek information and advice on diet from reputable sources'<br>**1 2 3 4 5 6 7 8 9 10**
(g) 'Dispel any myths about what and how much to eat during pregnancy'<br>**1 2 3 4 5 6 7 8 9 10**
(h) 'Offer practical and tailored information. This includes advice on how to use Healthy Start vouchers to increase the fruit and vegetable intake of those eligible…'<br>**1 2 3 4 5 6 7 8 9 10**

Thank you for completing the pre course questionnaire we hope you enjoy participating in the training today.
