# Supplementary material for: Eating for 1, Healthy and Active for 2; feasibility of delivering novel, compact training for midwives to build knowledge and confidence in giving nutrition, physical activity and weight management advice during pregnancy
Source: BMC Pregnancy Childbirth. 2014 Jul 4;14:218. doi: 10.1186/1471-2393-14-218 (PMC4227285; doi:10.1186/1471-2393-14-218)
Supplement: Additional file 2 — Questionnaire (Post training). Study tool designed to determine changes in self reported knowledge and confidence of midwives on the topic of nutrition, physical activity and weight management after attending the compact training, and to elicit satisfaction with the training received. [file 1471-2393-14-218-S2.pdf]

Questionnaire (Post Training)

1. **After participating in the course today, what would you say about your <u>knowledge</u> of the topics areas below?**
   For each question please tick the option that best suits you

(a) The range of risks related to obesity in pregnancy	My knowledge:<br><br>Is much better ( )<br>Is somewhat better ( )<br>Is a little better ( )<br>Has stayed the same ( )
(b) Pregnancy specific food and nutrition messages (based on the eatwell plate)	My knowledge:<br><br>Is much better ( )<br>Is somewhat better ( )<br>Is a little better ( )<br>Has stayed the same ( )
(c) Vitamins recommended during pregnancy, particularly for women with a raised BMI (including why, when, and amounts)	My knowledge:<br><br>Is much better ( )<br>Is somewhat better ( )<br>Is a little better ( )<br>Has stayed the same ( )
(d) The benefits of being physically active during pregnancy	My knowledge:<br><br>Is much better ( )<br>Is somewhat better ( )<br>Is a little better ( )<br>Has stayed the same ( )
(e) Recommended weight gain for women during pregnancy	My knowledge:<br><br>Is much better ( )<br>Is somewhat better ( )<br>Is a little better ( )<br>Has stayed the same ( )
(f) Ways to initiate conversations with women about ‘change’ related to their dietary and physical activity behaviours	My knowledge:<br><br>Is much better ( )<br>Is somewhat better ( )<br>Is a little better ( )<br>Has stayed the same ( )

2. After participating in the course today what would you say about your <u>confidence</u> to deliver on each of the following statements (extracted from NICE guidance 27, 2010)?
For each question please tick the option that best suits you

(a) 'Measure weight and height at the first contact with pregnant women'	My confidence:<br><br>is much better ( )<br>is somewhat better ( )<br>has stayed the same ( )<br>is somewhat worse ( )<br>is much worse ( )
(b) '… being sensitive to any concerns she [the woman] may have about her weight'	My confidence:<br><br>is much better ( )<br>is somewhat better ( )<br>has stayed the same ( )<br>is somewhat worse ( )<br>is much worse ( )
(c) 'Explain to women with a booking appointment BMI of 30 or more how this poses a risk, both to their health and the health of the unborn child'	My confidence:<br><br>is much better ( )<br>is somewhat better ( )<br>has stayed the same ( )<br>is somewhat worse ( )<br>is much worse ( )
(d) 'Explain that they should not try to reduce this risk by dieting while pregnant and that the risk will be managed by the health professionals caring for them during their pregnancy'	My confidence:<br><br>is much better ( )<br>is somewhat better ( )<br>has stayed the same ( )<br>is somewhat worse ( )<br>is much worse ( )
(e) 'At the earliest opportunity...discuss her eating habits and how physically active she is. Find out if she has any concerns about diet and the amount of physical activity she does and try to address them'	My confidence:<br><br>is much better ( )<br>is somewhat better ( )<br>has stayed the same ( )<br>is somewhat worse ( )<br>is much worse ( )
(f) 'Advise that a healthy diet and being physically active will benefit both the woman and her unborn child during pregnancy....Advise her to seek information and advice on diet from reputable sources'	My confidence:<br><br>is much better ( )<br>is somewhat better ( )<br>has stayed the same ( )<br>is somewhat worse ( )<br>is much worse ( )

(g) 'Dispel any myths about what and how much to eat during pregnancy'	My confidence:<br><br>is much better ( )<br>is somewhat better ( )<br>has stayed the same ( )<br>is somewhat worse ( )<br>is much worse ( )
(h) 'Offer practical and tailored information. This includes advice on how to use Healthy Start vouchers to increase the fruit and vegetable intake of those eligible…'	My confidence:<br><br>is much better ( )<br>is somewhat better ( )<br>has stayed the same ( )<br>is somewhat worse ( )<br>is much worse ( )

3. Did you learn anything about food and nutrition that you had not know before
Yes ( ) No ( )
If yes please give one or two examples

____
____
____

4. Was there anything, which was not included in the course, which should have been?
If yes what was this?____
____
____

5. Overall how would you rate the course (please tick one only)

Excellent ( )
Good ( )
Adequate ( )
Poor ( )

6. Would you recommend the course to others?

Yes ( ) No ( )

Please use the space below to comment on any other aspects of the training:

Thank you for completing the evaluation forms this will help to identify how valuable the training has been and whether any improvements can be made. As part of the evaluation process I will contact you in 3-6 months time to find out how the course has helped to support you, and your patent/ client group.
